# On the Influence of Freight Trains on Humans: A Laboratory Investigation of the Impact of Nocturnal Low Frequency Vibration and Noise on Sleep and Heart Rate

**DOI:** 10.1371/journal.pone.0055829

**Published:** 2013-02-07

**Authors:** Michael G. Smith, Ilona Croy, Mikael Ögren, Kerstin Persson Waye

**Affiliations:** 1 Occupational and Environmental Medicine, The Sahlgrenska Academy at the University of Gothenburg, Gothenburg, Sweden; 2 The Swedish National Road and Transport Research Institute, Gothenburg, Sweden; The Ohio State University, United States of America

## Abstract

**Background:**

A substantial increase in transportation of goods on railway may be hindered by public fear of increased vibration and noise leading to annoyance and sleep disturbance. As the majority of freight trains run during night time, the impact upon sleep is expected to be the most serious adverse effect. The impact of nocturnal vibration on sleep is an area currently lacking in knowledge. We experimentally investigated sleep disturbance with the aim to ascertain the impact of increasing vibration amplitude.

**Methodology/Principal Findings:**

The impacts of various amplitudes of horizontal vibrations on sleep disturbance and heart rate were investigated in a laboratory study. Cardiac accelerations were assessed using a combination of polysomnography and ECG recordings. Sleep was assessed subjectively using questionnaires. Twelve young, healthy subjects slept for six nights in the sleep laboratory, with one habituation night, one control night and four nights with a variation of vibration exposures whilst maintaining the same noise exposure. With increasing vibration amplitude, we found a decrease in latency and increase in amplitude of heart rate as well as a reduction in sleep quality and increase in sleep disturbance.

**Conclusions/Significance:**

We concluded that nocturnal vibration has a negative impact on sleep and that the impact increases with greater vibration amplitude. Sleep disturbance has short- and long-term health consequences. Therefore, it is necessary to define levels that protect residents against sleep disruptive vibrations that may arise from night time railway freight traffic.

## Introduction

Sleep is one of the most important sources for regeneration of the body and “needs its integrity to allow the living organism to recuperate normally” [1]. Disturbed sleep can therefore be of consequence for immediate and long term health [2]. In today’s society a multitude of sources exist with the potential to disturb sleep, one of the most prevalent being environmental exposure from transportation. In a well-controlled Japanese study involving 3600 women, the participants reported around a threefold enhanced risk for insomnia when they lived in high vs. low traffic exposure areas [3].

The adverse effects of noise on sleep are well studied and guidelines have been established [4], but there is a lack of research and understanding of how vibration influences sleep. This question is of high environmental importance, especially as there is an expected increase in the market share of freight traffic from 8% in 2001 to 15% in 2020 [5]. To contextualise the potential problem, recent field studies have observed between 3–19 nightly freight pass-bys in Norway [6] and up to 150 during bedtime in Germany [7].

A great deal of research has investigated the effect of traffic noise on the subjective quality and physiological character of sleep. Previous studies have primarily analysed the effects of railway [8], [9], [10], aeroplane [11], [12], [13], [14], [15] and road [16], [17], [18], [19] traffic noise. In summary there is clear evidence of sleep disturbance caused by night time traffic (for overview see [1], [2], [20]). Night time traffic noise has been shown to lead to fragmentation of sleeping patterns [11], [18], [21], cardiovascular changes [22], [23] and increased awakenings [11], [15] throughout the night. Such sleep disturbance can result in secondary effects such as poor sleep quality [24], fatigue, somnolence and reduced mental and physical functioning during day time [25], [26], [27]. This corresponds with reduced cognitive performance [20] after experimental nights with nocturnal traffic.

Because of these well documented effects the World Health Organization recommends a nocturnal bedroom noise level upper limit of *L*_Aeq,8h_ = 30dB and *L*_AF,max_ = 45dB [5]. However, no such recommendation exists for vibration, nor do these guidelines specifically account for low frequency components of the noise. For traffic induced vibration caused by railways for instance, freight trains typically have heavier axle loads than passenger trains, resulting in generation of higher amplitude low frequency vibration. The issue is compounded with the fact that soft ground conditions such as clay are particularly sensitive to low frequency propagation [28]. Measurements in the field also show that airborne noise from freight has increased energy in the lower frequencies, which may be important for sleep disturbance [29]. Given the current lack of research towards the effect of low frequency vibration on sleep, and since the nightly time slots are expected to play an important role in facilitating the envisaged increase of goods traffic, it is therefore advantageous to investigate its potential impact. Each passing train causes noise, which is audible and vibration, which is palpable. In field studies it is often reported to be difficult to differentiate if sleep disturbance is caused by the noise or the vibration character of a passing train.

Only a limited number of studies methodically examine the influence of vibration on sleep. A study by Arnberg and colleagues [30] systematically investigated the influence of vibration on sleep. Nine participants slept in a laboratory and were subjected to 140 vibration exposures of 2s duration in both the vertical and horizontal directions simultaneously, simulating heavy road traffic. Five of the participants were exposed to different vibration levels and 4 were exposed to a combination of noise and vibration. Although the small number of participants must be borne in mind, the study revealed that sleep is more disturbed from a combination of road traffic noise and vibration than from noise alone. It was also shown that sleep quality (both subjectively rated and measured as amount of REM-sleep) decreased, as did performance in the morning.

Laboratory studies investigating the effects of vertical vibration on sleep were also performed as part of the Swedish TVANE project [31], [32]. It was found that self-reported sleep disturbance was greater for high vibration amplitude, irrespective of noise level. A decrease in subjective sleep quality was observed when increasing the vibration amplitude from 0.4 to 1.4 mm/s (Nordic comfort weighted [33]). Although these trials included vibration typical of freight trains during the exposure nights, additional noise events for high speed and commuter trains were included. Since they used vertical excitation whole body vibration was found to be dependent upon subject mass. Additionally, only two vibration amplitudes with no intermediate value were used and the investigation was based solely upon subjectively reported data. As such it is felt that additional work incorporating physiological measurements, improved equipment and focussing specifically on variations arising from different vibration amplitudes is of significant usefulness.

In this study we aim to determine the impact of nocturnal freight train induced vibration and low frequency noise on subjective sleep quality, subjective sleep disturbance and heart rate. Based on the literature we hypothesise an increase of sleep disturbance with increasing vibration amplitude. The results will also assist in providing guidance for establishing acceptable levels of vibration and vibration induced noise from railway transportation during night time as defined within the remit of the EU CargoVibes project [34].

## Methods and Materials

Twelve volunteers (7 males, 5 females, mean age 22.3 s.d. ±2.5, range 20–29) slept for 6 consecutive nights in the laboratory. An initial habituation night was followed by a control night and by four randomised order exposure nights. In the exposure nights 36 train pass-bys were simulated, with varying vibration level (noise alone, low, moderate, high vibration) between nights. Great care was exercised to simulate realistic environmental conditions with the highest possible ecological validity. Methods are described in detail below.

### Ethics Statement

The study followed the Declaration of Helsinki on Biomedical Research Involving Human Subjects and was approved by the Ethics Committee from the University of Göteborg (920-11). All participants provided written informed consent.

### Vibration and Noise Exposure

On-site facilities comprised of three identical rooms furnished to simulate a home bedroom environment. For noise exposure in each room eighty eight 10″ speakers mounted within the ceiling panels reproduced the low frequency component of noise, and two corner loudspeakers handled the high frequencies. The crossover frequency between both systems was 125 Hz. Background noise level within the rooms was below 14 dBA. As part of the living quarters a privately accessed communal area equipped with cooking and washing facilities, a dining area, television and seating was provided for study subjects during their participation.

Previous investigations have either focussed on vertical excitation [35] or combined vertical and horizontal vibration [30]. Bedrooms are typically located on upper floors of dwellings, and horizontal vibration increases as a function of floor number [36] so it may be supposed that horizontal vibrations will subsequently be more important when evaluating sleep disturbance. Beds are generally placed along the edges of rooms and vertical vibration tends to dominate only in the centre of floors [37]. Previous work [38] investigating vibration along the *x* and *z* axes [39] found that for comparable amplitudes of whole body vibration measured on the body, elicited annoyance was higher in the horizontal direction. Vibration was therefore applied in the horizontal direction (*z*-axis, head – foot) using electrodynamic transducers rated at 1000W affixed to the underside of the bed frame (frequency response 5–40 Hz, driven by separate power amplifiers).

Five train passages were synthesised based upon analysis of accelerometer and sound level measurements performed in the field. Acoustic data for each train is presented in Table 1 and time histories are presented in Figures S1–S6. Each exposure night comprised of 36 synthesised train passages with a combination of high and low intensity hours. The periods of 23∶00–01∶00 and 05∶00–07∶00 were of higher passage frequency than 01∶00–05∶00 to reflect typical real world timetabling (see Table S1). Noise signals were filtered to correspond to a closed window. Background ventilation noise of 25 dBA was introduced into the bedrooms for the duration of the trial, including during the habituation and control nights.

**Table 1:** Vibration and noise parameters applied to individual trains.

	Noise exposure				Vibration exposure (same for all trains)	
Train	*L*_AEq_ (dB)	*L*_AF,max_ (dB)	*t* >35 dB (s)	T_10%–90%_ (s)	Unweighted acceleration(m/s^2^ rms)	W_d_ Weighted max. acceleration(m/s^2^)
1	44.0	48.4	11.5	8.9		
2	42.7	47.2	46.2	9.8	High = 0.072	High = 0.0204
3	44.5	49.8	23.7	8.4	Moderate = 0.036	Moderate = 0.0102
4	45.6	49.8	29.2	7.9	Low = 0.020	Low = 0.0058
5	42.4	47.2	56.9	9.2		

**Figure 1:**
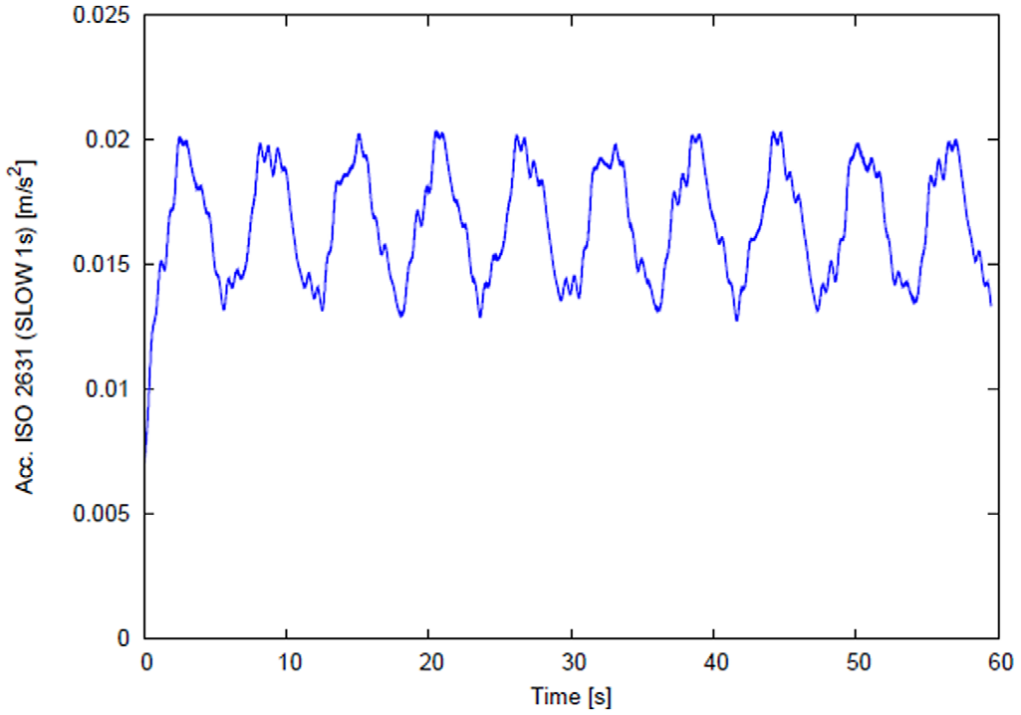
Amplitude modulated vibration reference signal adjusted to level and length for each train. Illustrated signal peaks at 0.0204 m/s^2^, corresponding to the High vibration exposure condition. Vibration rise time was identical for all vibration conditions.

The vibration pattern was identical between trains, independent of noise envelope but synchronised with the start and end times, with duration *t* for each train adjusted to values in Table 1. Vibration excitation began when *L*_AEq_ >35 dB, continuing for duration *t*. Vibration amplitude of presented stimuli was varied across exposure nights in a Latin square with participants being blind to the exposure. A 10 Hz vibration signal was amplitude modulated with the signal *c*(*t*).
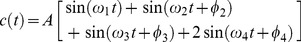
(1)where angular frequency *ω_n_* = 2*πf_n_* with *f_n_* being the modulation frequency and *φ_n_* is phase.



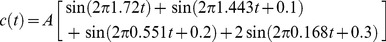
(2)Amplitude *A* was adjusted to give W_d_ weighted [39] acceleration values listed in Table 1. The weighted reference vibration signal with peak amplitude corresponding to high vibration exposure is presented in Figure 1.

When excited sinusoidally at 10 Hz along the *z*-axis, vibration measured on the frame of an unloaded bed in the laboratory is dominated by the 10 Hz driving frequency. Low amplitude harmonics are evident, but the highest in amplitude was a factor of 50 below the 10 Hz component.

### Questionnaires

To assess sleep disturbance during the experimental nights, separate morning and evening questionnaires were developed to record subjective sleep indicators, as well as tiredness and stress. The morning questionnaires were completed immediately following the 7∶00 awakening and comprised of questions on sleep quality, rated on an 11-point graded scale with endpoints *Very good* to *Very bad*, and on a 5-grade semantic scale composed of the wordings *Very good*, *Good*, *Not particularly good*, *Poor* and *Very poor*. Questions were posed on their current feeling, with *Very rested* - *Very tired*, *Very relaxed* - *Very tense* and *Very irritated* - *Very glad* as anchors on 11-point numerical scales. Also included were questions pertaining to sleep onset time and number of awakenings. Other questions on the subject’s experience of the night and sleep included difficulty in falling asleep, perception of how their sleep compared to normal and whether sleep was deep or shallow. Questions assessing sleep disturbance resulting directly from vibration and noise exposure followed with semantic grades of *Not at all*, *Hardly at all*, *Somewhat*, *Very* and *Extremely*. At the end were questions on stress/energy [40]. The evening questionnaire was administered within the 15 minute period prior to lights out at 23∶00, and included questions on tiredness, alertness and stress/energy.

### Polysomnography

Sleep parameters were obtained through use of polysomnography (PSG). All electrode placements and recordings were performed according to pertinent AASM standards [41]. Heart rate activity was recorded during the night time period through use of a single modified electrocardiograph (ECG) Lead II using torso electrode placement. Electroencephalogram (EEG), electrooculogram and submental electromyogram were recorded using surface electrodes. Blood oxygen saturation, pulse and plethysmogram were recorded using a finger pulse oximeter. Breathing rates were recorded using thorax and abdomen piezoelectric effort belts. Data was recorded onto an ambulatory PSG device (SOMNOscreen plus PSG+, SOMNOmedics GmbH, Germany). Recordings were analysed using DOMINO 2.4.0 (SOMNOmedics GmbH, Germany). Owing to unforeseen technical limitations, there were occasions where the quality of recorded EEG data was not suitable over the entirety of an individual night to perform in-depth analysis of sleep staging (for instance, fluctuating impedance values). Therefore a conservative approach was taken, and EEG recordings were used only to identify artefacts and wake stages to be excluded from heart rate analysis. This means heart rate events were excluded where either artefacts in the sleep stage were present, or where wake stages were observed having been automatically scored with a high degree of accuracy using good quality signals.

### Participants

The twelve volunteers were recruited by public advertising. They were screened to ensure they did not use tobacco products, were not caffeine dependent, maintained normal sleeping habits, were free of sleeping problems, did not snore or suffer from breathing difficulties and were not on regular medication affecting sleep. To minimise mass-loading effects and respiratory problems during sleep it was required they have a BMI <28 (mean BMI 21.6 s.d. ±2.3 range 19–27.4, mean mass 68.25 kg s.d. ±7.46). Subjects were prohibited from caffeine consumption after 15∶00 and sleeping at times other than the exposure period of 23∶00–07∶00 to ensure sleep was not influenced by external factors other than the experimental stimuli. In order to ascertain normal hearing, each participant was required to pass an audiometric test with hearing assessed to a threshold of 20 dB HL at frequencies of 0.25, 0.5, 1, 2, 4 and 8 kHz.

Prior to the habituation night subjects assessed their individually rated normal sleep quality, sensitivity to noise and tolerance to vibration through application of a questionnaire. Ten of the participants rated their home bedroom environment as either very or fairly quiet. Nine reported themselves as not particularly sensitive and 3 as quite sensitive to noise. Eight described themselves as somewhat tolerant and 4 as very tolerant to vibration. It must be noted that these descriptors were not part of the selection criteria.

### Statistical Analysis

Data was analysed using SPSS v. 18 (SPSS Inc., Il., USA). Questionnaire data was analysed using ANOVA for repeated measurements with post-hoc Bonferroni-corrections to account for multiple testing. To investigate the effect of exposure, analyses included the control night. Specific analysis of vibration amplitude included only the four exposure nights. For event related changes of heart rate, the 10s preceding each noise stimulus exceeding 35 dB was calculated as a baseline value and compared with the 60s following onset of each exposure. To ensure a solid baseline value, all within-night exposures were combined and an average calculated over all events (36 trains) per night. Previous studies indicate that heart rate response to traffic noise differs depending on whether subjects wake up or continue sleeping [42]. As such, the few events in which the participants were awake (a 30 second epoch automatically scored as wake stage by the analysis software, manually cross-checked to ensure reliability of the scoring) prior to train onset or awoke within 1 minute of train onset were excluded from the calculation. The amplitude and latency of maximum change of heart rate within the first 30s after train onset was identified per night for each participant. In order to identify overall change of heart rate, the integral of the curve (AuC) was calculated. A level of α = 0.05 was considered statistically significant.

## Results

### Rating Data

**Figure 2:**
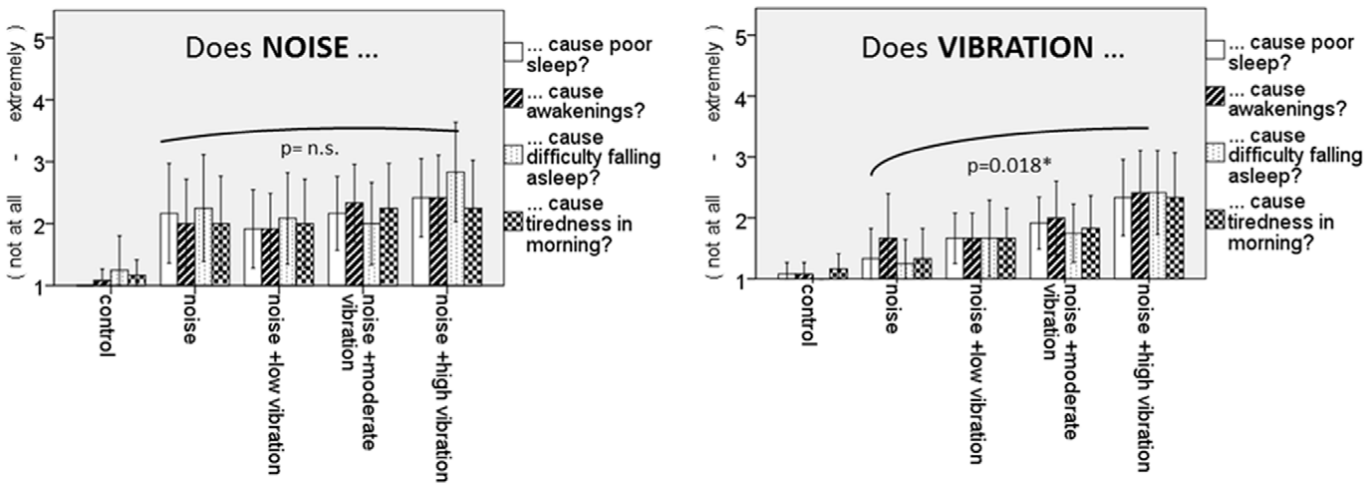
Subjectively rated poor sleep, awakenings, difficulty falling asleep and tiredness in the mornings. Left panel indicates response when rating noise exposure as the cause. Right panel indicates response when rating vibration exposure as the cause. No significant differences due to noise are observed with increasing vibration amplitude. Poor sleep, difficulty falling asleep and tiredness in the mornings due to vibration increases with increasing vibration amplitude.

Results from the morning questionnaires are presented in Table 2. A linear effect was observed for overall subjective sleep quality decreasing significantly with an increase of vibration level (p = 0.033, F(3,7) = 6.1). Additionally, a linear tendency was observed as a function of exposure including the control night (p = 0.055, F(4,6) = 4.7). With increasing vibration amplitude, participants rated to feel significantly more disturbed by vibrations (p = 0.002, F(3,8) = 16.2). Including the control night the linear effect reached higher significance (p<0.001, F(4,7) = 30.6). Participants combined ratings of differentiated effects of vibration on sleep parameters also showed clear influence of applied vibration amplitude (p = 0.018, F = 3.87, Figure 2). This effect was significant in each of the individual subscales of poor sleep, difficulty to fall asleep, tiredness in the morning (each p<0.05), but not for awakenings. In contrast, participants rating of being disturbed by noise did not change significantly with increasing vibration amplitude (p = 0.626, F(3,7) = 0.253). In the evening, participants rated to feel more stressed with increase of vibration level the night before (p = 0.048), although this effect did not survive Bonferroni-correction for multiple measurements. Other measurements of sleep revealed no statistically significant effect of exposure or vibration amplitude.

**Table 2:** Sleep parameters subjectively assessed using the questionnaire administered in the mornings.

	Control		Noise only		Noise & low vibration		Noise & moderate vibration		Noise & high vibration		ANOVA
Subjective parameter	Mean	s.d.	Mean	s.d.	Mean	s.d.	Mean	s.d.	Mean	s.d.	
Sleep quality (Very good = 10, very bad = 0)	5.8	2.4	6.2	2.0	6.8	2.2	5.2	1.8	4.3	2.2	p_vib_ = 0.033 p_exp_ = 0.055
Sleep disturbance by vibrations (0 = Not at all,10 = extremely)	0.4	0.9	1.1	2.1	2.3	1.7	3.3	1.9	4.7	2.7	p_vib_ = 0.002 p_exp_<0.001
Sleep disturbance by noise (0 = Not at all,10 = extremely)	0.7	1.8	4.6	3.0	3.6	2.7	4.2	2.4	4.7	2.5	n.s.
Rested (0)–Tired (10)	4.3	2.2	3.9	2.3	3.7	2.3	4.2	2.1	5.3	2.5	n.s.
At ease (0)–Tense (10)	2.6	0.9	3.2	1.6	2.9	1.6	3.8	1.8	4.3	2.0	n.s.
Glad (0)–Irritated (10)	2.7	1.6	2.3	1.4	2.3	1.4	2.5	1.9	3.9	1.9	n.s.
Time to fall asleep (minutes)	19.2	9.0	29.6	19.4	27.9	15.0	24.6	12.0	27.1	14.7	n.s.
Estimated number of wakeups	2.0	1.3	2.5	1.3	2.1	1.3	2.8	2.0	3.3	2.9	n.s.
Easy to sleep (0)–Difficult to sleep (10)	3.1	2.3	4.6	2.7	4.7	2.6	4.5	2.2	5.3	2.3	n.s.
Slept better than usual (0)–Slept worse than usual (10)	5.2	1.7	5.5	1.6	5.8	1.5	5.7	1.8	6.8	1.3	n.s.
Slept deep (0)–Slept light (10)	3.5	1.4	4.3	1.9	3.8	1.7	4.3	1.5	5.3	2.3	n.s.
Never woke (0)–Woke often (10)	4.6	2.1	4.9	2.0	4.8	1.8	5.3	2.0	5.3	2.7	n.s.
Stress	1.3	0.5	1.4	0.5	1.4	0.7	1.4	0.6	1.7	0.9	n.s.
Energy	2.7	0.7	2.8	0.8	2.8	0.7	2.8	0.6	2.6	1.0	n.s.

p values of ANOVA for repeated measurements are presented for increasing the vibration amplitude (p_vib_) and for analysis including the control night (p_exp_).

### Heart Rate

**Figure 3:**
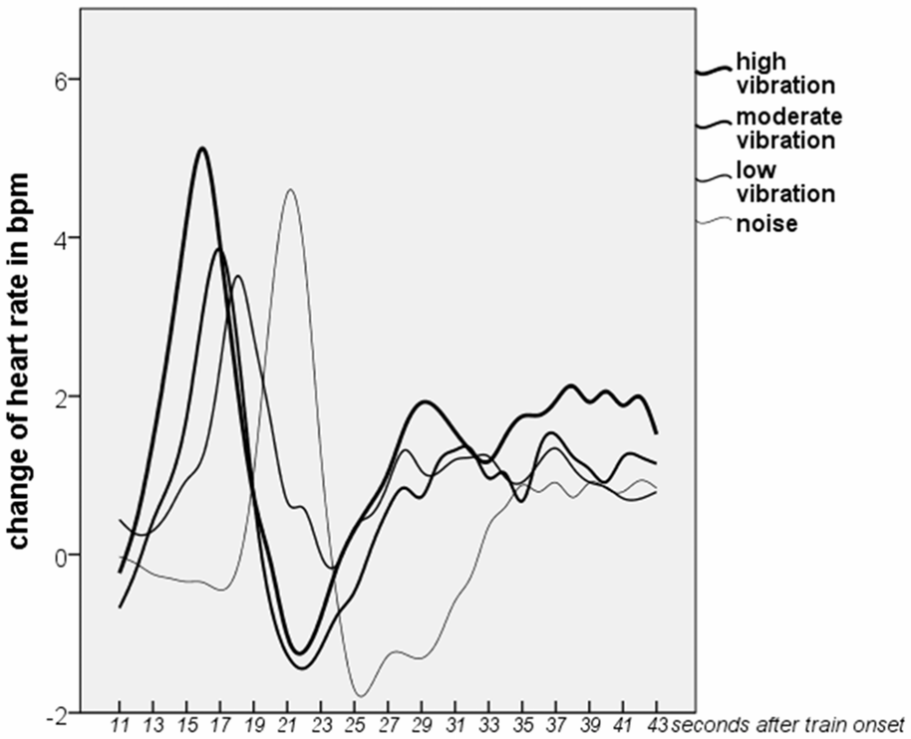
Changes in heart rate response and latency averaged for each of the four vibration exposure conditions. The increase in heart rate is significantly higher in the high vibration and noise condition than in the noise only condition. The heart rate increase latency is inversely related to vibration amplitude.

Excluding observed awakenings and artefacts, train induced changes of heart rate could be identified for 6 participants in noise only, 9 in the low, 7 in the moderate and 10 in the high vibration conditions. Figure 3 illustrates changes of heart rate compared to the baseline in the four experimental conditions. ANOVA revealed a linear increase of heart rate increase onset time with increasing vibration amplitude (p = 0.043, F(3,4) = 8.6). Furthermore, a tendency of an overall increase of heart rate (AuC) was observed with increasing vibration amplitude (p = 0.054, F(3,4) = 7.3).

## Discussion

The aim of this study was to investigate the impact of nocturnal vibration and low frequency noise arising from freight trains upon cardiovascular response and subjective sleep parameters. Sleep disturbance and low sleep quality [24] can lead to fatigue, somnolence and reduced mental and physical functioning during day time following exposure nights [25], [26], [27]. It was initially hypothesised that sleep quality and disturbance would be detrimentally affected by increases in vibration amplitude. The observed results of reduced sleep quality, enhanced disturbance and increase of heart rate generally support this hypothesis.

With increasing vibration exposure from low to high, we found a significant reductive effect in overall subjective sleep quality, agreeing with the few studies that have systematically investigated the impact of vibration on sleep [30], [31], [32]. We observed no significant differences between sleep quality in the noise only and low vibration conditions, again supporting previous work from Öhrström et al [32] where no sleep disturbance from vibration was observed at 0.4 mm/s (weighted according to the Nordic standard [33]). The comparatively poor sleep quality reported after the control night in relation to the exposure nights is worth some consideration. As the control night was always the second night of the trial, immediately following the habituation night, the sleep quality during the control night could be negatively affected if subjects did not habituate fully during the habituation night. Previous research has shown that a single night of habituation is very likely adequate for laboratory PSG experiments on sleep [43] but it has also been suggested that reduced sleep quality during adaptation may result in carry over effects for the second night although such an effect would be expected to be small [11]. If the control night was negatively affected this could lead to an underestimation of the effects that we noted, and would be worth following up in future studies.

Similar patterns are observed in the results of sleep disturbance due to vibration, which increases monotonically with increasing vibration amplitude. This is in agreement with an experimental study which showed that a large proportion of people have their sleep disturbed at high vibration values [32]. It additionally found that noise demonstrates no significant contribution to the perceived disturbance due to vibration i.e. there is an absence of an interaction effect when assessing vibration. The current study design maintains an equal noise exposure across experimental nights so it is not possible at this time to determine any interactions, and if so whether there is an antagonistic effect, with assessment of vibration being reduced with concurrent noise, or a synergistic effect where vibration assessment is increased alongside noise.

We found no significant differences in sleep disturbance due to noise across all exposure nights. This is not an unexpected result since noise exposure was identical for each exposure night. However, taking into account the observed rise in disturbance with increasing vibration amplitude as the cause, it suggests that participants were able to correctly identify the impact of each stimulus.

Greater vibration amplitudes were found to have a significant impact on perceived poor sleep, lead to greater difficulty falling asleep and cause more tiredness in the mornings. No significant effects were observed when asked to report noise as the cause. This again supports the suggestion that individuals are able to distinguish between exposures.

Sleep is known to be important for maintaining good health, and it is negatively affected by awakenings and disruption [2]. It is therefore desirable wherever possible to minimise external exposures that may impact upon disturbance and fragmentation. Freight train noise has been shown to be particularly disruptive on sleep, causing an increased number of awakenings compared with passenger trains and aircraft [8], [44]. Alongside other rail transit, freight trains are generally of greater length which increases the pass-by duration. If such an event leads to an awakening, the possibility of it subsequently being consciously perceived and hence recalled the following morning may therefore be greater [20]. Events perceived in such a way perhaps then have a large influence on subjective sleep quality, as has been proposed previously [45]. Prior to now, the influence of low frequency vibration specifically from freight has been little investigated, and given the increase in sleep disturbance and decrease in sleep quality we observed additional research is certainly required to evaluate the human impact.

An increase in heart rate was observed following the onset of exposure, followed by a deceleration to below the baseline. This biphasic response is in agreement with earlier observations for transportation noise exposure [23]. Following heart rate response induced by noise, the heart rate returns to a value slightly above baseline [42] as is also seen in our results for the noise only condition. The introduction of vibration subsequently corresponds to a higher heart rate following recovery. The overall increase in heart rate (AuC) is higher in the high vibration condition compared with the noise only exposure night. The effect was just over the required level of significance, very likely because of the small number of participants. However, the effect size is rather high. As heart rate response does not habituate across nights [42], vibration exposure may be important for the impact on the cardiovascular system. As described in the methods, events where participants awoke were excluded events from analysis. Griefahn et al [42] showed differences in HR reaction to traffic noise with and without awakenings. The adopted approach allows a very focussed and conservative estimation of the HR response. However, as HR reactions are usually much higher in terms of awakenings, our results likely underestimate the effects.

Changes in heart rate may be an indicator for long term cardiovascular health. As pointed out by Babisch [16] there is evidence that exposure to transportation noise can lead to hypertension and ischaemic heart disease in the long term. In particular he notes the positive association between hypertension and subjectively reported sleep disturbance. The increased disturbances with higher vibration amplitudes in our results along with the changes in heart rate suggest that a similar connection between the potential risk for certain cardiovascular diseases and exposure to high vibration amplitudes may be present.

There was also a linear increase in latency inversely related to the magnitude of vibration exposure. Although this response has been reported for noise previously [10], [23], [46], [47], [48], to the author’s knowledge this is the first time the relationship has been demonstrated for vibration exposure. For noise exposure, sound pressure rise time has been shown to be an important determinant for rate of acceleration in heart rate [20], [42]). It is unclear whether this is also the case for vibration as by design the vibration exposure rise time in our trial was identical between trains and across exposure amplitudes. Our data at least suggests that for vibration the intensity is also of great importance. The noise exposure without exception began prior to the vibration so it is possible that heart rate response is influenced by interactions between both exposures, supported by the increased latency of response in the noise only condition compared with vibration exposure nights. Any such interactions certainly offer interesting avenues for further research.

The authors are aware of the limitations of the study, which shall now be addressed in point. The trial was conducted in a laboratory environment although efforts were made to ensure high ecological validity. Despite significant observed effects on sleep with increasing vibration, the relatively low number of participants (N = 12) and the focussed age group means that further work is required to generalise the conclusions drawn to a wider population. The sample size calculation was based on field results of sleep disturbance caused by traffic noise [19]. However, according to the classification of Cohen only large effects are detected and small or mediums effects are likely missed by the study [49]. Heart rate response and heart rate amplitude responses to nocturnal noise for instance have previously been shown to be age dependent [10], where the cardiovascular response was greater in young compared with middle aged subjects. Since our trial used young healthy subjects, the applicability of the results to a wider population should be treated accordingly.

Vibration exposure has also been considered. For recumbent subjects such as those lying in bed, whole-body vibration is independent of their body position, meaning each individual experienced the same vibration during the course of the exposure nights [50], and neither is the mass of each participant expected to have a large influence [38]. Vibration was applied in the horizontal direction only rather than also in the vertical plane as vertical excitation is known to be highly attenuated by the mattress [30].

The impact of individual trains may be an issue. Different noise rise times have previously been shown to affect heart rate response [20]. In our study each of the five trains had a different rise time, but due to the relatively limited number of individual trains per night (eight occurrences each for trains 1, 2, 3 and 4 and four occurrences of train 5) further reduced by the exclusion of events where either artefacts or awakenings occurred, heart rate reactions were analysed by combining all exposures within each trial night rather than investigating the five separate trains individually. We can hence not draw conclusions regarding the impacts of the individual train’s characteristics, including rise time and event duration. A greater number of events during the night would allow for sufficient numbers of each train to allow a more detailed analysis of the influence of the characteristics of each.

### Conclusions

Our study shows that individuals are able to differentiate between train induced vibration and train induced noise during the night and that train induced vibration and low frequency noise has a negative effect on their self-reported sleep quality, causes subjective sleep disturbance and is accompanied by heart rate increase. The effects increase with greater vibration amplitude. The results suggest that individuals living near to railway lines and thus subjected to the accompanying noise and vibration exposure are at risk for having their sleep impaired. This may lead to reduced concentration and daytime functioning in the short term and impaired health in the long term. Since night time railway freight traffic is expected to increase, mitigation measures towards reducing internal vibration amplitudes should therefore be taken in order to protect populations against potential adverse health effects.

## Supporting Information

Figure S1 **Noise time history for Train 1.**
(TIF)

Figure S2 **Noise time history for Train 2.**
(TIF)

Figure S3 **Noise time history for Train 3.**
(TIF)

Figure S4 **Noise time history for Train 4.**
(TIF)

Figure S5 **Noise time history for Train 5.**
(TIF)

Figure S6 **Third octave band frequency spectra of all trains.** Signal is filtered to correspond to a closed window, attenuating the high frequency components.
(TIF)

Table S1 **Distribution of train events and corresponding noise levels for each exposure hour.**
(DOC)
