# Supplementary figures and images for: On the Influence of Freight Trains on Humans: A Laboratory Investigation of the Impact of Nocturnal Low Frequency Vibration and Noise on Sleep and Heart Rate
Source: PLoS One. 2013 Feb 7;8(2):e55829. doi: 10.1371/journal.pone.0055829 (PMC3567002; doi:10.1371/journal.pone.0055829)

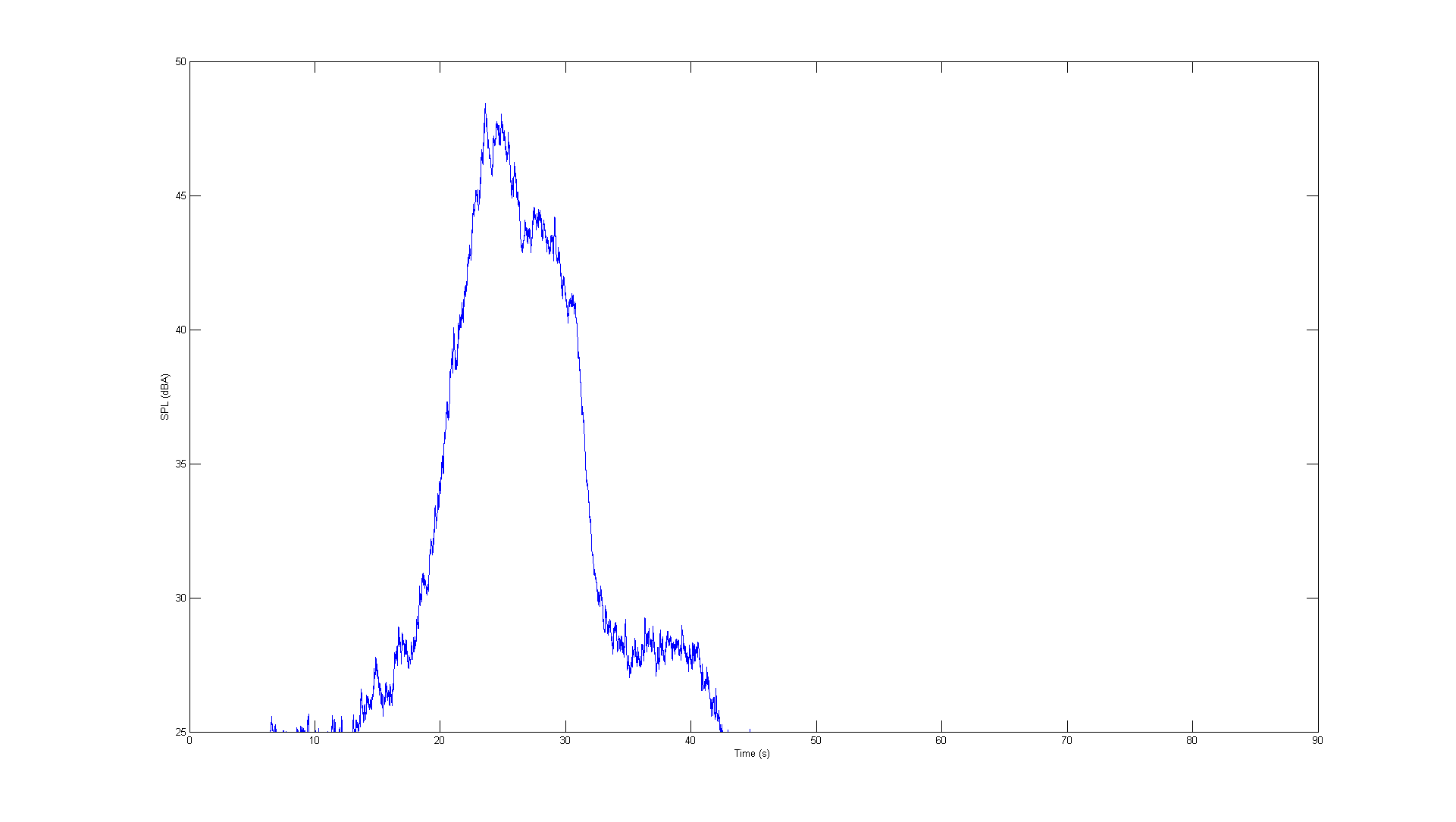

Supplement: Figure S1 — Noise time history for Train 1. (TIF) [file pone.0055829.s001.tif]

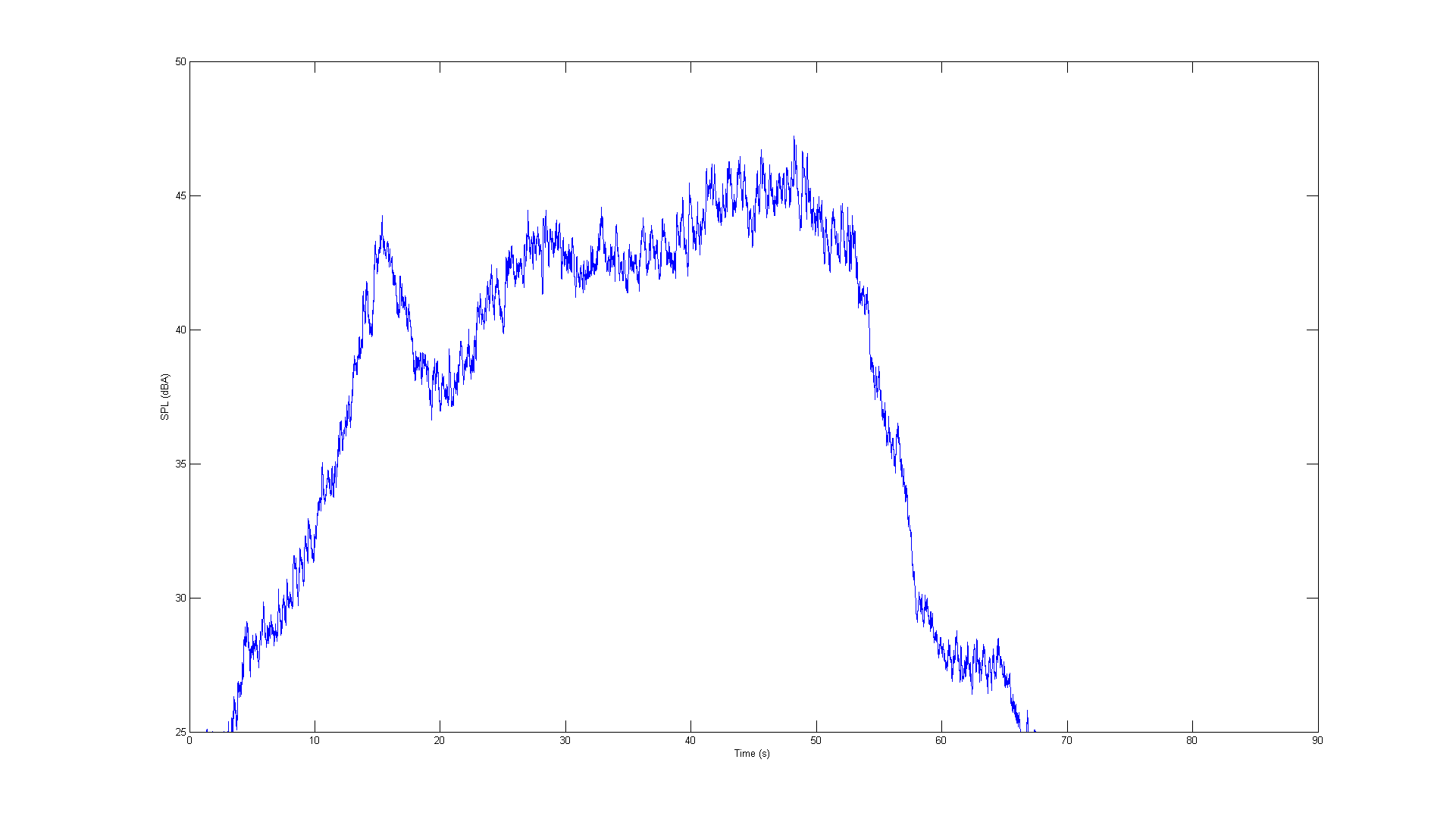

Supplement: Figure S2 — Noise time history for Train 2. (TIF) [file pone.0055829.s002.tif]

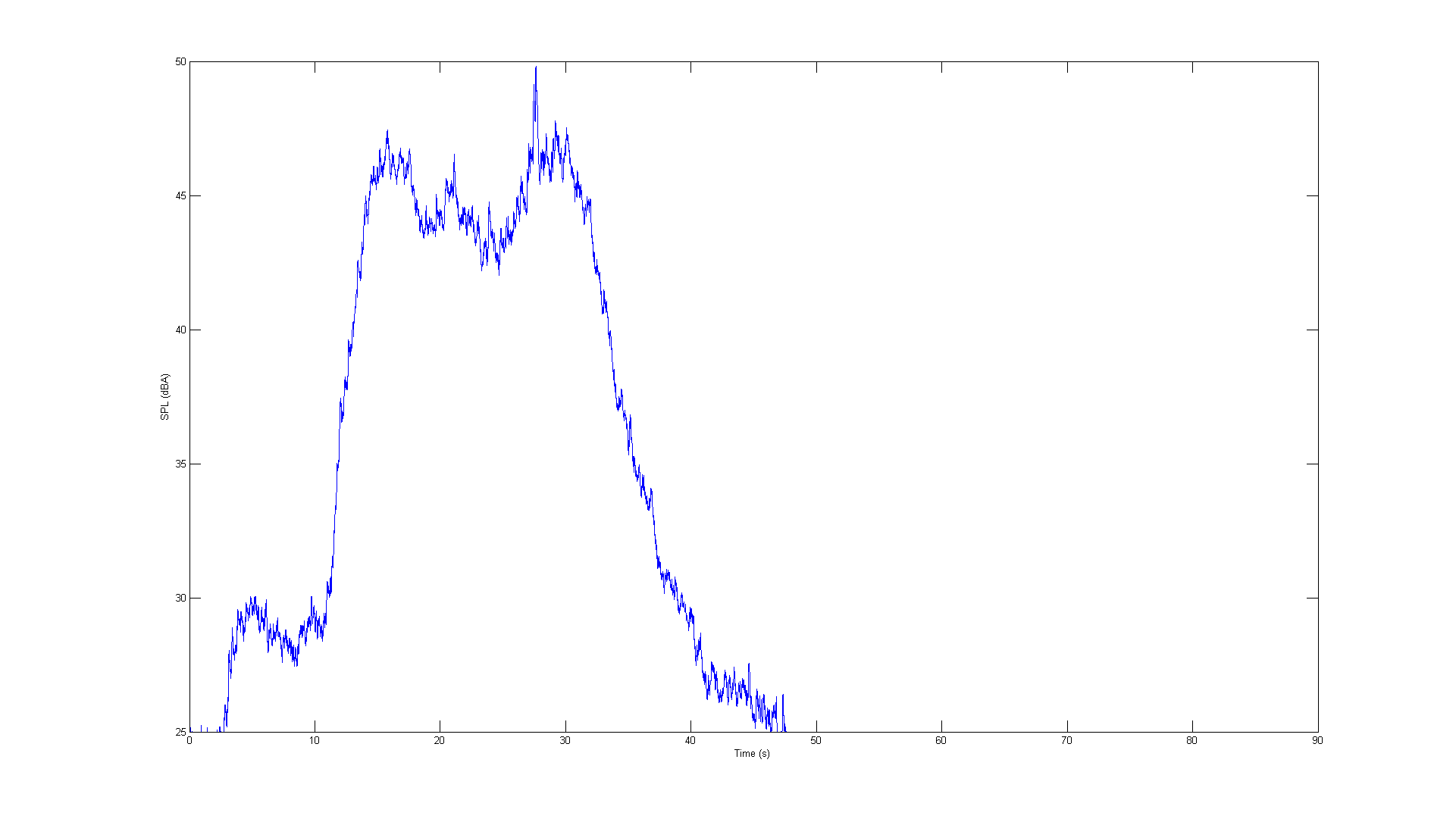

Supplement: Figure S3 — Noise time history for Train 3. (TIF) [file pone.0055829.s003.tif]

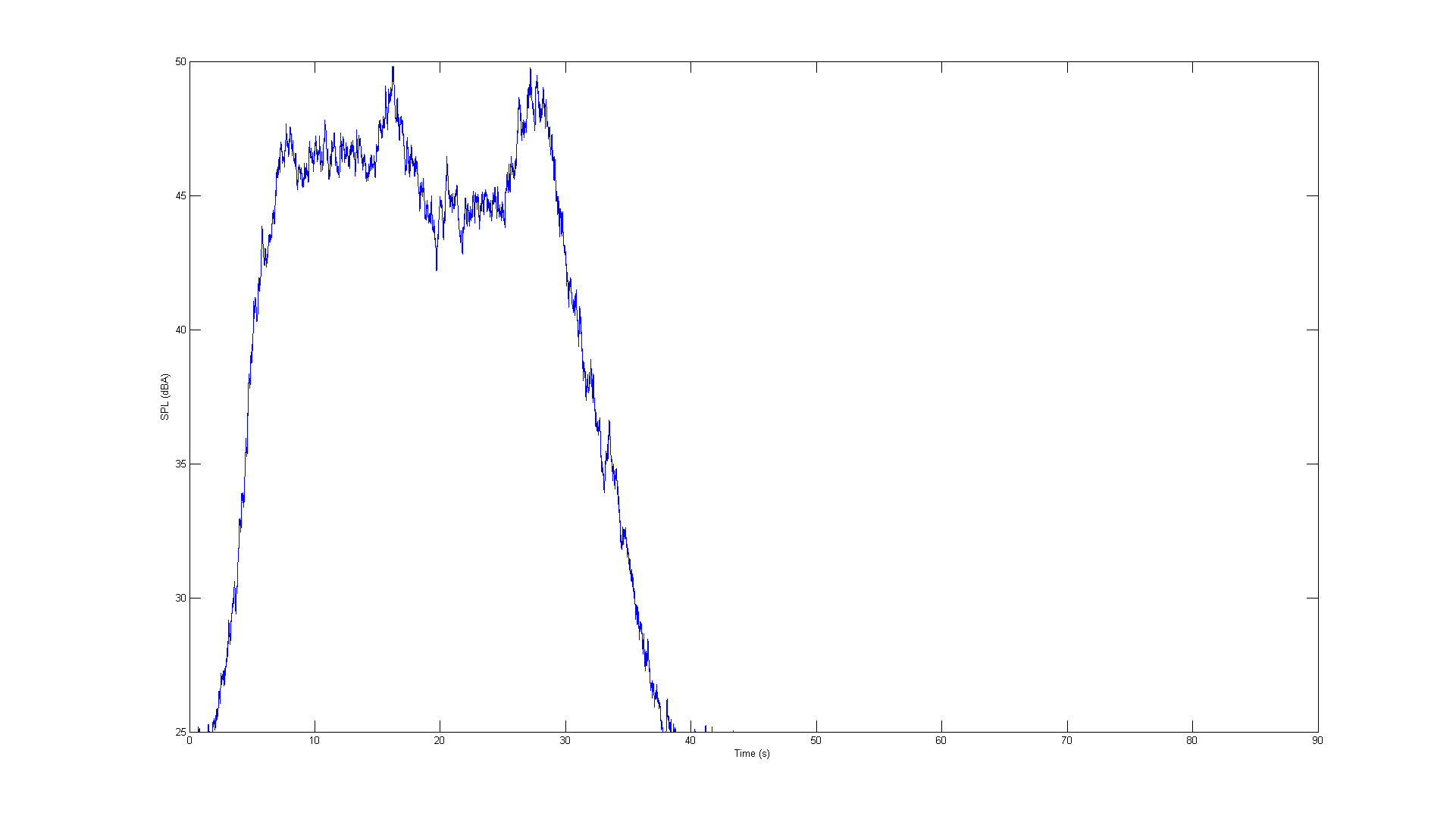

Supplement: Figure S4 — Noise time history for Train 4. (TIF) [file pone.0055829.s004.tif]

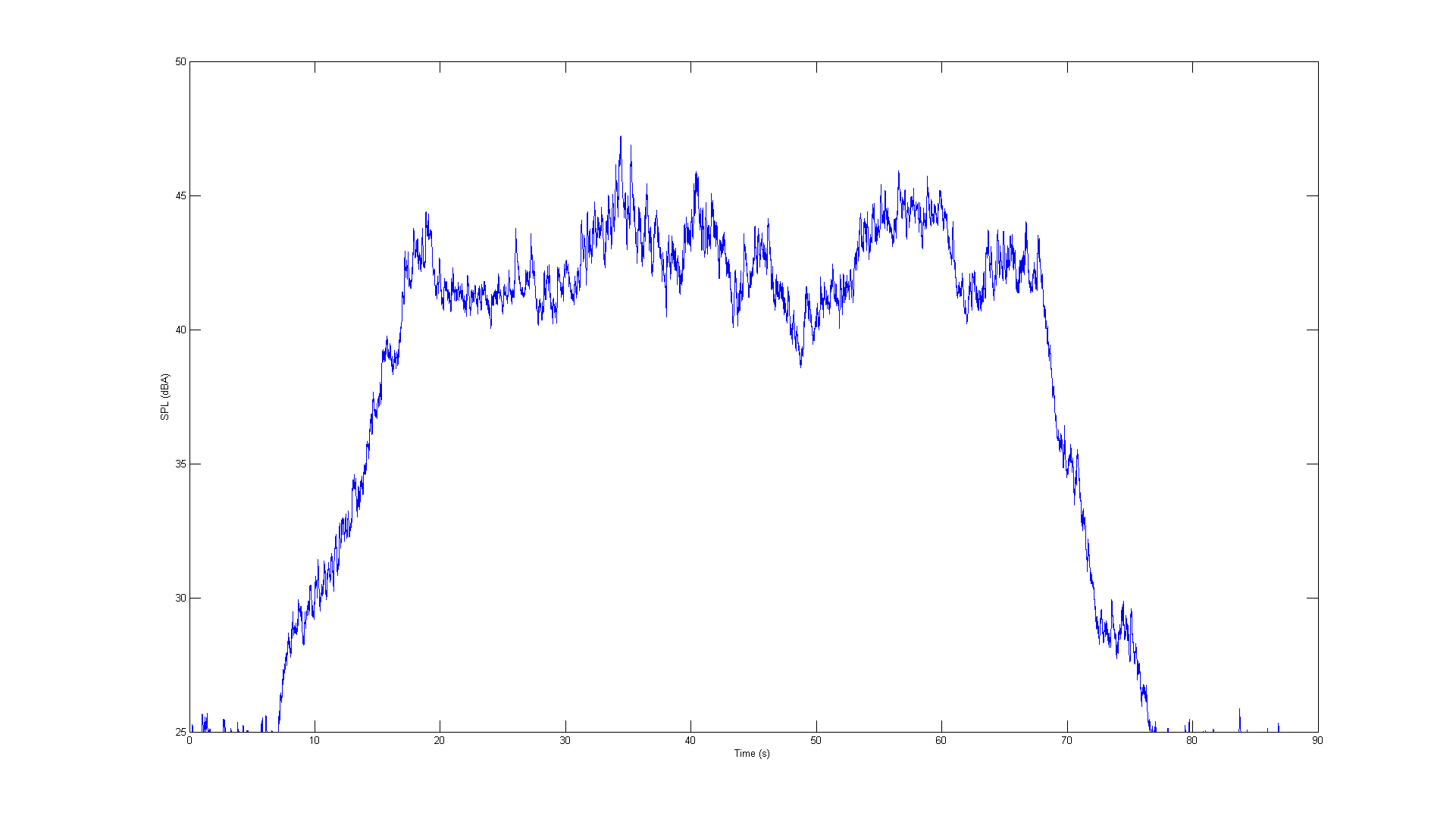

Supplement: Figure S5 — Noise time history for Train 5. (TIF) [file pone.0055829.s005.tif]

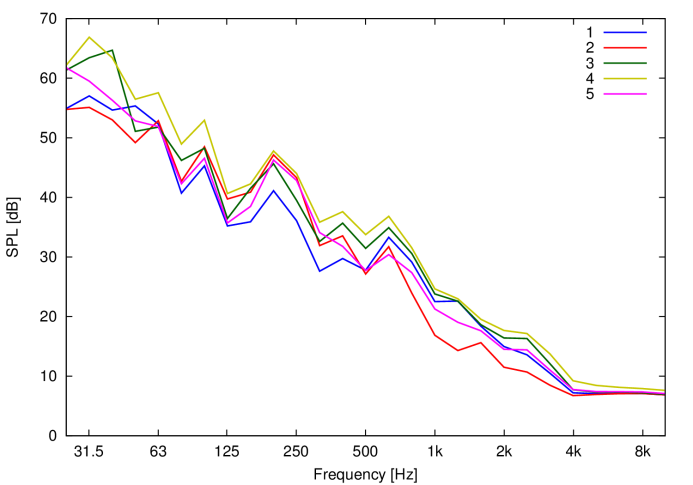

Supplement: Figure S6 — Third octave band frequency spectra of all trains. Signal is filtered to correspond to a closed window, attenuating the high frequency components. (TIF) [file pone.0055829.s006.tif]
