# Supplementary material for: On the Influence of Freight Trains on Humans: A Laboratory Investigation of the Impact of Nocturnal Low Frequency Vibration and Noise on Sleep and Heart Rate
Source: PLoS One. 2013 Feb 7;8(2):e55829. doi: 10.1371/journal.pone.0055829 (PMC3567002; doi:10.1371/journal.pone.0055829)
Supplement: Table S1 — Distribution of train events and corresponding noise levels for each exposure hour. (DOC) [file pone.0055829.s007.doc]

Table S1. Distribution of train events and corresponding noise levels for each exposure hour.

| Time | Train 1 | Train 2 | Train 3 | Train 4 | Train 5 | *L*_AEq,1h_ (dB) | *L*_AFmax_ (dB) |
| --- | --- | --- | --- | --- | --- | --- | --- |
| 23-00 | 1 | 1 | 1 | 2 | 1 | 32.3 | 49.8 |
| 00-01 | 1 | 1 | 1 | 2 | 1 | 32.3 | 49.8 |
| 01-02 | 1 | 1 | 1 | 0 | 0 | 29.1 | 49.8 |
| 02-03 | 1 | 1 | 1 | 0 | 0 | 29.1 | 49.8 |
| 03-04 | 1 | 1 | 1 | 0 | 0 | 29.1 | 49.8 |
| 04-05 | 1 | 1 | 1 | 0 | 0 | 29.1 | 49.8 |
| 05-06 | 1 | 1 | 1 | 2 | 1 | 32.3 | 49.8 |
| 06-07 | 1 | 1 | 1 | 2 | 1 | 32.3 | 49.8 |
| 23-07 | 8 | 8 | 8 | 8 | 4 | *L*_AEq,8h_ (dB)  31.3 | 49.8 |
